# Novel Bloch wave excitation platform based on few-layer photonic crystal deposited on D-shaped optical fiber

**DOI:** 10.1038/s41598-021-90504-z

**Published:** 2021-05-28

**Authors:** Esteban Gonzalez-Valencia, Ignacio Del Villar, Pedro Torres

**Affiliations:** 1 Escuela de Física, Universidad Nacional de Colombia - Sede Medellín, A.A. 3840 Medellín, Colombia; 2 Department of Electronic and Telecommunications Engineering, Instituto Tecnológico Metropolitano, Medellín, Colombia; 3 Institute of Smart Cities (ISC), Public University of Navarra, 31006 Pamplona, Spain; 4 Electrical and Electronic Engineering Department, Public University of Navarra, 31006 Pamplona, Spain

**Keywords:** Optical sensors, Photonic devices, Metamaterials

## Abstract

With the goal of ultimate control over the light propagation, photonic crystals currently represent the primary building blocks for novel nanophotonic devices. Bloch surface waves (BSWs) in periodic dielectric multilayer structures with a surface defect is a well-known phenomenon, which implies new opportunities for controlling the light propagation and has many applications in the physical and biological science. However, most of the reported structures based on BSWs require depositing a large number of alternating layers or exploiting a large refractive index (RI) contrast between the materials constituting the multilayer structure, thereby increasing the complexity and costs of manufacturing. The combination of fiber–optic-based platforms with nanotechnology is opening the opportunity for the development of high-performance photonic devices that enhance the light-matter interaction in a strong way compared to other optical platforms. Here, we report a BSW-supporting platform that uses geometrically modified commercial optical fibers such as D-shaped optical fibers, where a few-layer structure is deposited on its flat surface using metal oxides with a moderate difference in RI. In this novel fiber optic platform, BSWs are excited through the evanescent field of the core-guided fundamental mode, which indicates that the structure proposed here can be used as a sensing probe, along with other intrinsic properties of fiber optic sensors, as lightness, multiplexing capacity and easiness of integration in an optical network. As a demonstration, fiber optic BSW excitation is shown to be suitable for measuring RI variations. The designed structure is easy to manufacture and could be adapted to a wide range of applications in the fields of telecommunications, environment, health, and material characterization.

## Introduction

Electromagnetic surface waves (ESWs) are widely studied due to their potential applications in photonic devices and sensing applications in areas such as biology, chemistry, physics, among others^1–3^. The first ESWs to be observed were the surface plasmon polaritons (SPPs), a type of resonance located at the interface between a metal film and a dielectric medium represented by the collective motion of electrons^4^. SPPs are characterized by their short propagation length, caused by the strong absorption of the metal layer^4,5^, which thus limits the scalability of such structures. Therefore, there is a high interest in surface waves between two dielectric media, since the ESWs can propagate at the interface with minimum losses^4,6^. Most of the proposed applications based on ESW excitation exploit the Kretschmann configuration, which is illumination through a bulky prism, at an angle greater than the critical angle of the multilayer. However, ESW excitation in optical fibers is considered as a more compact, lightweight and robust alternative, with potential for remote and live monitoring applications^1^, and for “lab-on-fiber” platforms that can be applied for communication and sensing purposes^7^.

Some of the most relevant ESWs in dielectric interfaces are the Bloch surface waves (BSWs)^8^, which consists of electromagnetic waves that propagate at the interface between two dielectric media, where at least one of them is periodically non-homogeneous in the normal direction to the interface^9^. In the simplest case, the periodically non-homogeneous medium consists of a one-dimensional photonic crystal (1DPC)^8,10^. Its confinement is due to total internal reflection from the side of the homogeneous dielectric and to the photonic bandgap in the 1DPC^11,12^. Thus, the BSW dispersion relation is below the light line of the homogeneous dielectric, and within the photonic bandgap^13,14^. Moreover, multilayers are wavelength scalable and may be designed to sustain TE- and/or TM-polarized BSWs at a broad range of wavelengths from near UV to IR^15^.

As a BSW propagates along the surface, it couples light to the multilayer system^16^. Thus, the multilayer design has been optimized for low losses by appropriately choosing the thicknesses and number of periods. Most devices based on BSW excitation require a 1DPC with a large number of layers–higher than 12^10,12,17–25^. These configurations have been used in 1DPCs consisting of bilayers of materials with refractive index (RI) differences as small as 0.2 refractive index units (RIU)^12,17–19^ up to moderate differences of 0.8 RIU^10,20–25^. However, there are reported studies that show that it is possible to excite BSWs at the interface of 1DPCs with less than 10 layers^5,26–31^. A common feature of these structures is the relatively large RI difference of the materials used in the 1DPC bilayers, ranging from values greater than 0.8 RIU^32,33^ to 2 RIU and even higher^5,31^. The resonant generation of BSWs via prims or grating coupling is an active field of current research in applications such as sensors^13,21,31,34,35^, surface-enhanced Raman spectroscopy^36,37^, fluorescence-based detection^29,38–40^, enhanced nonlinear effects^41,42^, and integrated optics^18,20^. However, very few investigations on the BSW excitation in optical fibers have been published, and not all of them have experimental verification^19,23,30,32,43^.

In this work, we report a BSW excitation platform that uses geometrically modified commercial optical fibers such as D-shaped optical fibers, with a few-layer 1DPC deposited on its flat surface using metal oxides with a moderate difference in RI. Indeed, it was recently demonstrated that D-shaped fibers can operate in reflective configuration^44^, which indicates that the structure proposed here can be used as a probe that can be used as a catheter, a nasogastric probe, or even for chemical mapping of surfaces. The combination of fiber–optic-based platforms with the nanotechnologies is opening the opportunity for the development of high-performance photonic devices that enhance the light-matter interaction in a strong way compared to other optical platforms. In this novel ESW excitation platform, BSWs are excited through the evanescent field of the core-guided fundamental mode. The 1DPC consist of alternating thin layers of tin oxide ($\hbox {SnO}_2$) and copper oxide (CuO), which have the real part of the RI higher than that of the pure silica, with which the commercial optical fibers are manufactured, and the imaginary part low but not negligible^45^. A high real part of the RI provides strong in-plane confinement of the light and enables the development of compact and low loss integrated photonic devices. The nano-deposition technique used for generating the 1DPC was DC sputtering and a theoretical analysis was made, which permitted to analyze and validate the experimental results. Although most of the BSW excitation platforms require a large number of layers or materials with a large difference in RI, we demonstrate that even a 3-layer stack on the fiber-based excitation platform can sustain TE-polarized BSWs using a 1DPC consisting of materials that have a moderate difference in RI. To the best of our knowledge, this constitutes the first experimental observation of BSWs excitation in laterally polished commercial optical fibers. Moreover, the 1DPC is easy to fabricate and the results are promising for the development of new types of all-fiber photonic devices and sensing applications.

## Results and discussion

### Design of the structure

**Figure 1:**
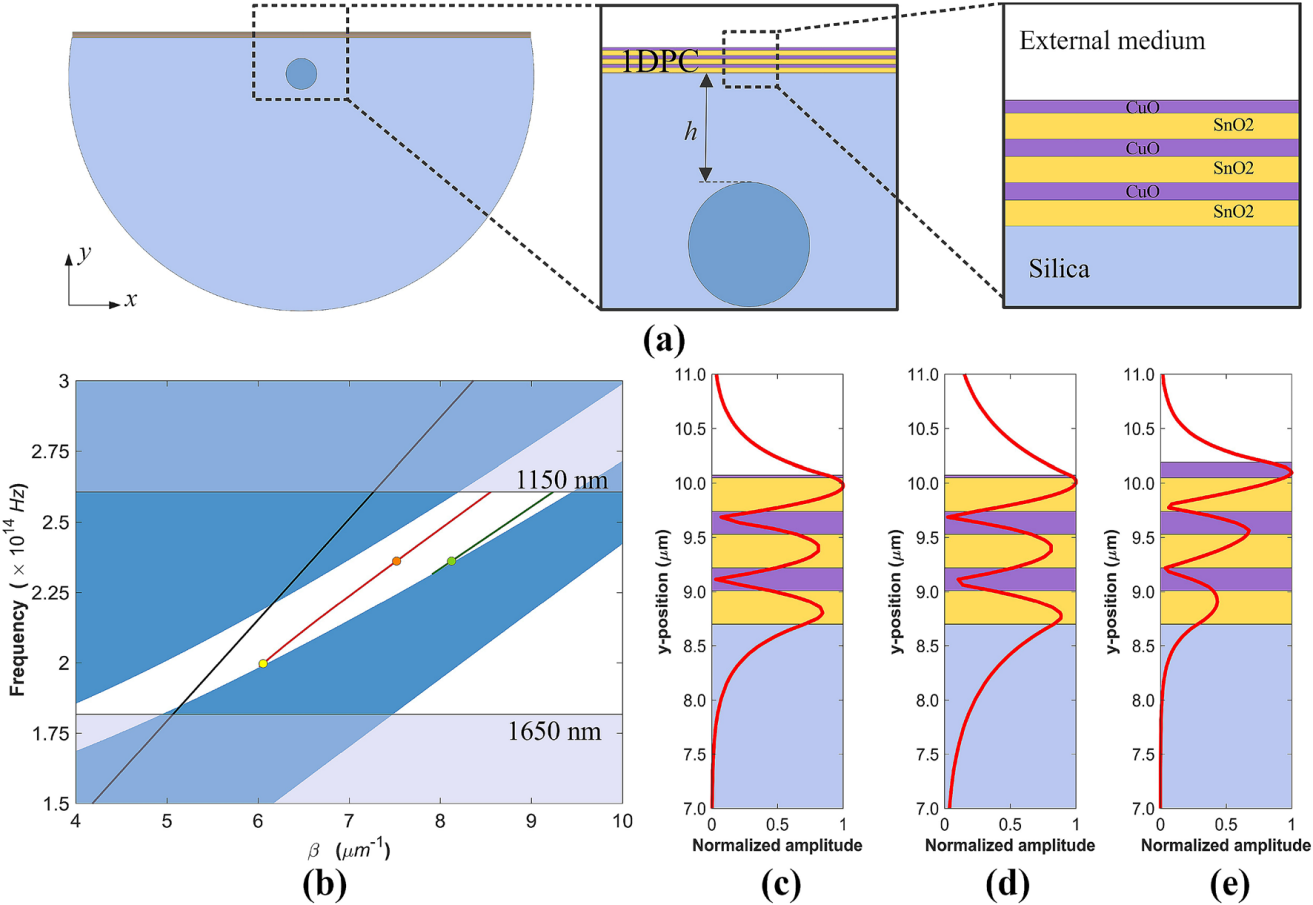
(**a**) Schematic of a single mode D-shaped fiber coated with a 6-layer 1DPC. The orange and purple layers represent the $\hbox {SnO}_2$ and CuO, respectively. (**b**) Band diagram of the $\hbox {SnO}_2$/CuO 1DPC for the TE polarization. The black line is for light in the surrounding medium (1.33), the red and green lines are the calculated BSW modes at the 6-layer 1DPC with a termination layer of 24 nm and 140 nm, respectively. (**c**)(**d**)(**e**) BSW electric-field intensity distributions at the 1DPC with termination layer of 24 nm [at 1270 nm (**c**), and 1500 nm (**d**)] and 140 nm [at 1270 nm (**e**)].

The structure designed to experimentally verify the excitation of BSWs in optical fiber consists of a commercially available single mode D-shaped fiber optic and a few-layer 1DPC coating the flat surface of the fiber, as shown in the schematic of Fig. 1a. In this figure, the two insets zoom the central region, where the multilayer deposited on the flat surface of the fiber can be identified. It is a structure that is easy to implement, manipulate, and allows to reduce associated costs^46^. There are many studies on thin-film depositions on geometrically deformed fibers, where the core-guided evanescent field can be coupled to waveguides^47^, surface gratings^48^, metal films to excite SPPs^46^, and metal oxides to generate lossy-mode resonances^49–52^, among others. The 1DPC consist of alternating thin layers of tin oxide ($\hbox {SnO}_2$) and copper monoxide (CuO) that exhibit a moderate RI contrast, close to 0.3 at the near infrared region (1.84 for $\hbox {SnO}_2$ and 2.137 for CuO at 1550 nm), and relatively low extinction coefficient values (0.01 for $\hbox {SnO}_2$ and 0.02 for CuO, which were selected to match the experimental results reported below).

Because the RI difference between the $\hbox {SnO}_2$ and the CuO thin films is moderate, the thickness of the alternating layers of the 1DPC are chosen to achieve optimum BSW excitation condition and low losses. To design multilayers that match the conditions for BSW, the thicknesses of the high and low refractive index layers *d* are calculated based on the forbidden transmission band at the desired resonance wavelength $\lambda$ of a 1DPC bandgap material^11^, $d=\lambda /(4n)$ , where *n* is the refractive index within the high and low refractive index layers. For example, using this expression for the wavelength $\lambda = 1550$ nm, it is found that the thicknesses of the $\hbox {SnO}_2$ and CuO are 212 nm and 182 nm, respectively. Since the resonance wavelengths of BSWs are determined by the thicknesses of the multilayer, BSWs in the NIR region can be achieved by varying the layer thicknesses. In the present work, the thicknesses of the $\hbox {SnO}_2$ and CuO layers are 300 and 200 nm, respectively, which were chosen so that the multilayer exhibits a photonic bandgap around the near infrared, which is the common range of operating wavelengths in fiber-optic communication components (1150–1650 nm), and a TE-polarized BSW can be excited. The properties of a BSW are more easily understood from the study of the dispersion diagram of the semi-infinite multilayer. Figure 1b shows the dispersion band diagram of the proposed $\hbox {SnO}_2$/CuO 1DPC for TE polarization, in which the radiative zones (blue) and the non-radiative zones (white) are distinguished for propagation within the multilayer stack. The figure also shows the light line (black) of the surrounding medium, which was taken to be 1.33. The dispersion band diagram of the semi-infinite multilayer was calculated with the transfer matrix method^53^. The $\beta$ axis is the propagation constant of the incident light, which is parallel to the surface of the multilayer structure, and the frequency axis corresponds to the wavelength range of interest. The surface modes occur in the white area that correspond to the photonic bandgap of the dispersion band diagram. This allows us to excite the BSW modes of the structure, whose propagation properties strongly depend on the 1DPC termination. In practice, however, we have to deal with a finite multilayer, such as the one depicted in Fig. 1a. The strict sense of bandgaps with well-limited edges should be reconsidered since with so few periods they are vaguely defined. Although it seems to be a problem with the position of some cases of surface waves because they appear outside the ideal gaps, one can adhere to the criteria of the evanescent character of the fields and the ability of these systems to guide energy along the surfaces^54^, which results in a loss of energy in the transmitted light. Dispersion curves and electric field distributions for the fundamental BSW (BSW1) were numerically calculated using the commercial software package FIMMWAVE (details on the parameters used for the simulations are given in the “Materials and methods” section). For demonstration purposes, Fig. 1b also shows the relation of the BSW1 dispersion curves in the photonic bandgap for different thicknesses of the termination layer—the CuO layer adjacent to the surrounding medium—of the 6-layer 1DPC outlined in the inset of Fig. 1a. It can be observed that the BSW1 mode dispersion curve with a 24 nm CuO termination layer (red line) appears near the upper and lower edges of the bandgap, whereas the BSW1 dispersion curve with a 140 nm CuO termination layer (green line) lies near the lower edge of the bandgap. The electric-field intensity profiles in Fig. 1c–e correspond, respectively, to the orange, yellow and green dots, on the dispersion curves of the BSW1 modes in Fig. 1b. As expected, the field is highly enhanced at each surface of the multilayer and has an exponentially decaying shape inside the crystal. Interestingly, the field distributions in Fig. 1c,d, to the best of our knowledge, are the first evidence that in a finite periodic system of alternating dielectric layers bounded asymmetrically by dielectric media, the surface modes of each boundary can interact and become coupled to a variable degree depending on both the truncation of the outer layers as the total thickness of the 1DPC (see supplementary material). It is worth noting that the excitation of coupled surface modes has been reported in dielectric–metal–dielectric structures to excite long-range surface plasmon polaritons^55,56^ and finite dielectric 1DPCs bounded by air^54^ and SiC gratings^57^. For the surface modes that occur near the center of the bandgap (red dot), the mode is more confined and the attenuation by the multilayer stack is stronger. For the modes close to the edges (yellow dot), the evanescent field penetrates much further into the structure and the attenuation is relatively weaker. Therefore, the fiber-based BSW platform can be designed based on the desired application. Strong field enhancement is needed for sensing and nonlinear applications, while modes with longer propagation length are required for integrated optics^16^.

Similar to the prism-based BSW excitation, the fiber-based BSW can be efficiently excited as long as the effective index of the core-guided mode coincides with that of the BSW^32^. Besides sensitivity, one of the main determining factors for the resonance performance is the absorption loss of the multilayer materials. Increasing such losses yields considerably broadened resonance (see supplementary material). The number of layers in the stack also determines the resonance width and depth depending on the material properties. From what we learned from the 6-layer stack discussed above, we reduced the number of layers and included 3- and 5-layer $\hbox {SnO}_2$/CuO stacks in the study. To have better knowledge of the BSW excitation phenomenon in the D-shaped fiber, theoretical simulations were performed using the commercial software package FIMMWAVE (details on the parameters used for the simulations are given in the “Material and method” section).

**Figure 2:**
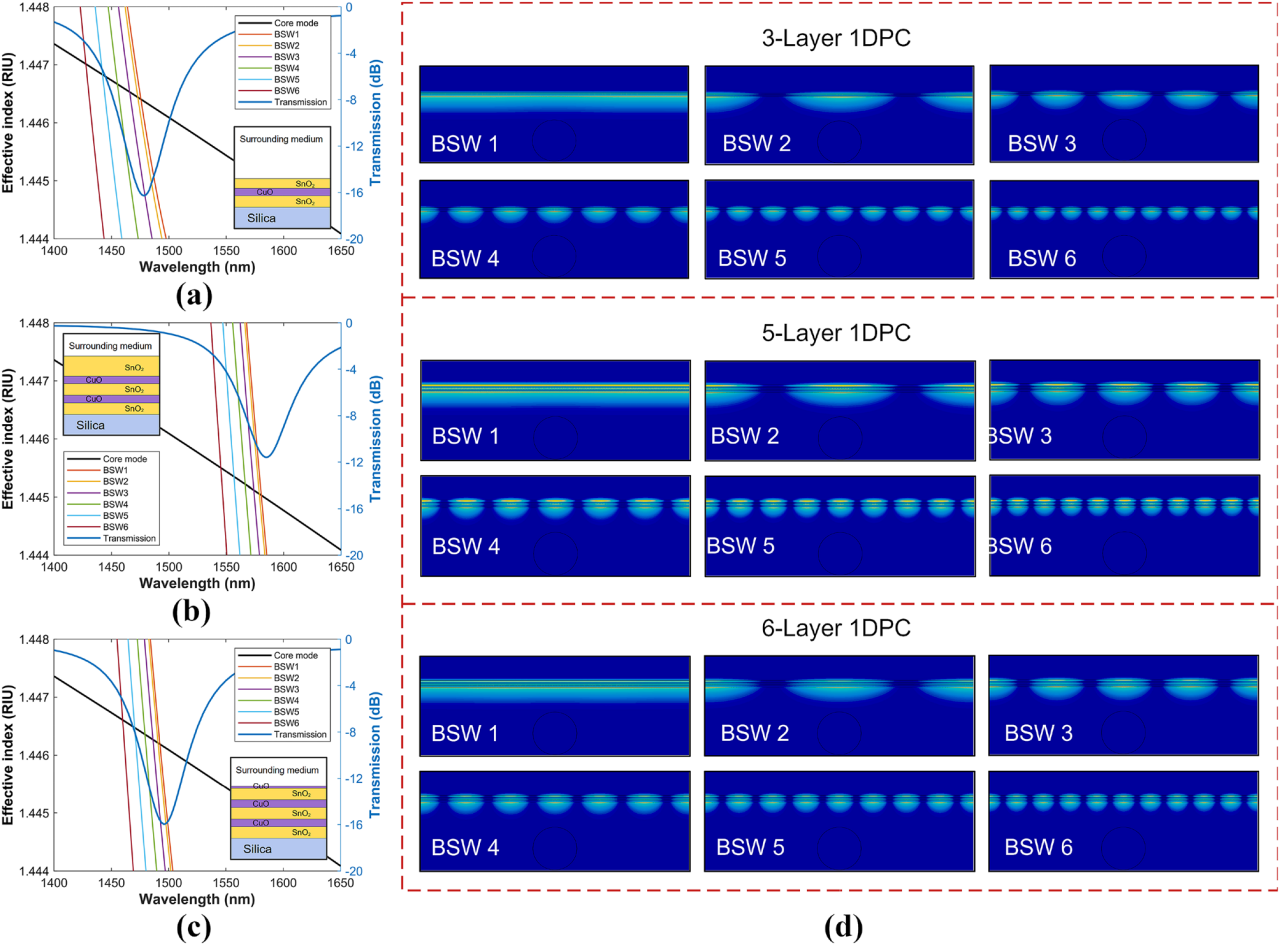
Dispersion curves of the core-guide mode and the BSW modes for the (**a**) 3-Layer, (**b**) 5-Layer, and (**c**) 6-Layer 1DPC. The blue lines (right axis) represent the calculated transmission spectra, and the figure insets are the 1DPC analyzed. (**d**) Optical field intensity distributions of the supported BSW modes for the analyzed 1DPCs. For the sake of simplicity, only the dispersion curves of the first 6 BSW modes are plotted.

Figure 2 shows the dispersion curves of the BSWs that can sustain the 3-, 5-, and 6-layer stacks designed in the spectral range of interest, with the thickness and material of the termination layer being 245 nm ($\hbox {SnO}_2$), 461 ($\hbox {SnO}_2$), and 50 nm (CuO), respectively, as outlined in the insets (the thicknesses were chosen so that the spectral position of the calculated spectra match with the experimental spectra, as will be shown later).

The phase matching conditions indicate that Bloch waves can be excited, and the power of the light can be efficiently transferred from the fiber core to the few-layer 1DPC. The structure can guide a finite number of BSW modes in a relatively narrow wavelength range. The number of guided modes, their transverse amplitude profiles and their propagation constants depend on the cross-sectional structure of the multilayer and on the optical frequency. In general, the resonance wavelength of a BSW mode of lower order occurs at a longer wavelength than a BSW mode of high order. For instance, the phase matching points for the 3-layer 1DPC are at 1475.6 (BSW1), 1473 (BSW2), 1465.9 (BSW3), 1455.2 (BSW4), 1442.3 (BSW5), and 1427.7 nm (BSW6); for the 5-layer 1DPC at 1580.5 (BSW1), 1578.8 (BSW2), 1574.0 (BSW3), 1566.4 (BSW4), 1556.7 (BSW5), and 1545.7 nm (BSW6); and for the 6-layer 1DPC at 1492.5 (BSW1), 1490.8 (BSW2), 1486.4 (BSW3), 1479.4 (BSW4), 1470.5 (BSW5), and 1460.0 nm (BSW6). It can be seen that the BSW phase-matching points of the 5- and 6-layer 1DPCs are closer together and the associated loss peaks in their transmission spectra of the core-guided mode (blue lines) are mostly caused by the attenuation bands of the surface modes BSW1 and BSW2 (for details see supplementary material). On the other hand, the phase matching points of the 3-layer 1DPC are more separated, making the loss peak in its transmission spectra wider since, in addition to the surface modes BSW1 and BSW2, the attenuation band of the BSW3 mode now has a higher contribution and, therefore, a higher light transfer from the fiber core to the 1DPC is observed in the transmission spectrum of the core-guided mode (see supplementary material). The optical-field intensity distributions in Fig. 2 show that the fields are highly confined to the termination layer and are concentrated within the 1DPC periods. This shows that 1DPCs can sustain different resonances depending on the dielectric properties of the multilayer stack.

### Fabrication and device characterization

**Figure 3:**
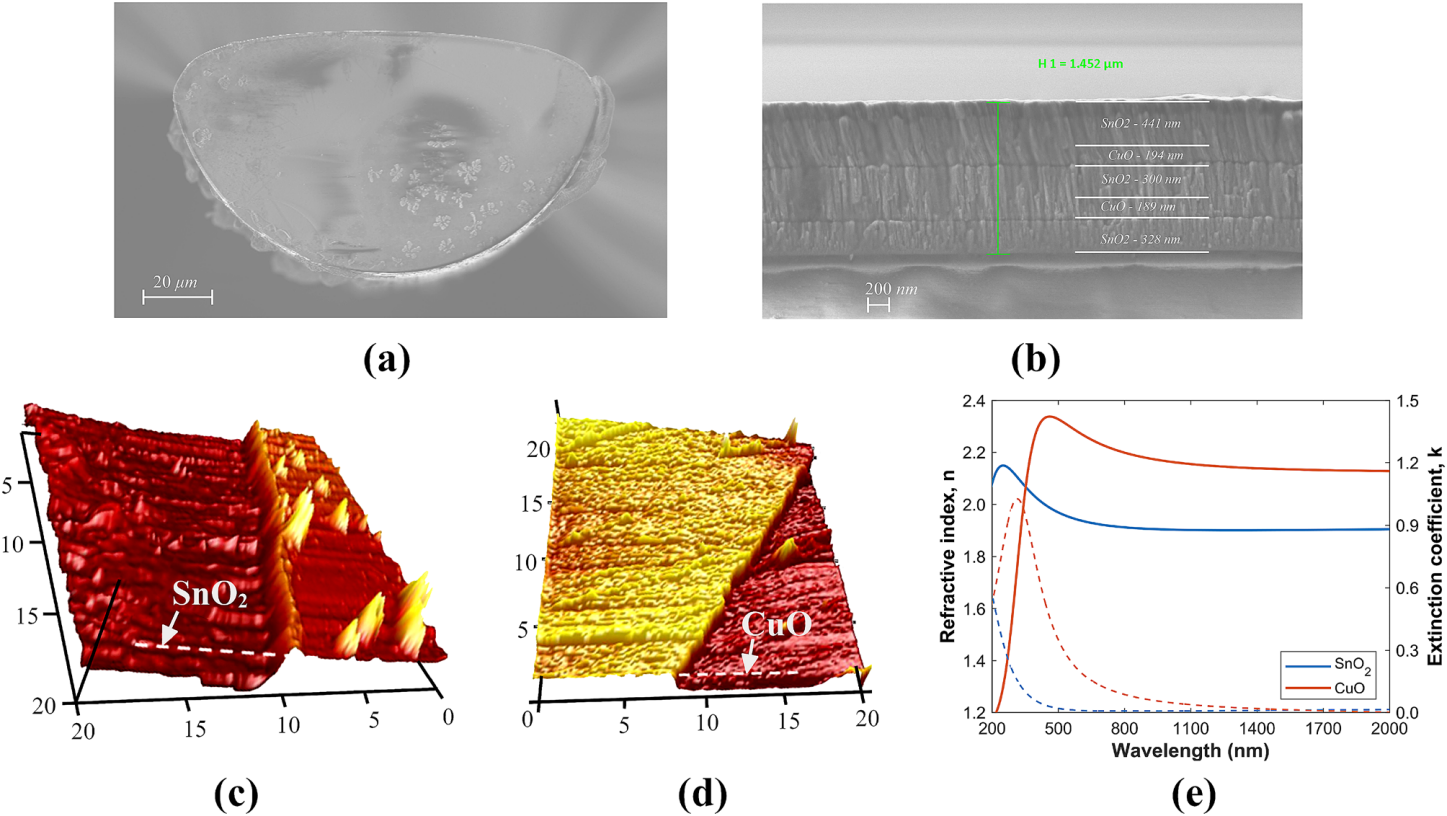
Characterization of coated D-shaped fiber with alternating layers of $\hbox {SnO}_2$ and CuO using a DC sputtering system. SEM images of the (**a**) D-shaped fiber cross-section with 5-layer 1DPC; (**b**) detail of the deposited 5-layer $\hbox {SnO}_2$/CuO 1DPC. AFM microscope images showing the roughness of the thin films deposited on coverslips for (**c**) $\hbox {SnO}_2$ and (**d**) CuO, of 75 nm and 36 nm thickness, respectively. (**e**) Refractive index (solid lines) and extinction coefficient (dashed lines) of the $\hbox {SnO}_2$ (blue) and CuO (red) thin films. (**c**-**e**) adapted with permission form Ref.^45^.

The three multilayer structures previously analyzed with the 3-, 5-, and 6-layer 1DPCs were fabricated using alternating $\hbox {SnO}_2$ and CuO on the flat surface of D-shaped standard fibers using a DC sputtering system (see “Materials and methods” section for more details). Note that in all cases, the first layer that is deposited on the flat surface of the D-shaped fiber is $\hbox {SnO}_2$, whereby the 1DPC termination layer depends on the number of layers. As an example, a scanning electron microscopy (SEM) image of the cross-section of the D-shaped fiber with 5-layer 1DPC is shown in Fig. 3a. The characterization of the fiber shows that the distance between the core and the polished surface of the D-shaped fiber is *h* = 4.5 $\mu$m. The SEM image in Fig. 3b allows to differentiate the profile of the metal oxide depositions, where the two $\hbox {SnO}_2$/CuO bi-layers can be clearly identified. From the analysis of the image it can be corroborated that the fabricated structure is very close to the designed multilayer stack of two $\hbox {SnO}_2$/CuO bi-layers of 300/200 nm and a $\hbox {SnO}_2$ termination layer of 461 nm (see inset in Fig. 2). In addition, thin films of $\hbox {SnO}_2$ and CuO deposited on coverslips placed at the same position of the fibers in the DC sputtering chamber were characterized with an atomic force microscope (AFM). A morphological study was performed in different zones in order to obtain the mean value of the film thickness and its surface roughness. The AFM images obtained are presented in Fig. 3c,d and show homogenous thin films of $\hbox {SnO}_2$ and CuO with average RMS roughness 4.7 nm and 2 nm, respectively. Furthermore, the thin films were also characterized with an ellipsometer to obtain the dispersion curves of the two materials. The wavelength dependence of the index of refraction and extinction coefficient of the $\hbox {SnO}_2$ and CuO thin films can be seen in Fig. 3e. The high (low) refractive index of the CuO ($\hbox {SnO}_2$) reveals the great potential of these metal oxides for the development of BSW platforms with moderate optical losses in the near-IR region of light. It is worth mentioning that in this work, extinction coefficient values of 0.01 for $\hbox {SnO}_2$ and 0.02 for CuO were chosen to match with the experimental results. Here it is important to explain that the angle of incidence of the ellipsometer was set to 70$^\circ$ because more accurate results are obtained for angles approaching the Brewster angle, and this value is recommended by the manufacturer, Horiba, for the metallic oxides that we have used in the experiment. Oppositely, light is guided in the fiber at grazing incidence (90$^\circ$) related to the thin-film, which leads to a higher light scattering. This is not considered by the ellipsometer. Consequently, we attribute to this factor the discrepancy between the extinction coefficient calculated by the ellipsometer and the extinction coefficient required to fit the BSWs observed in the optical spectrum.

**Figure 4:**
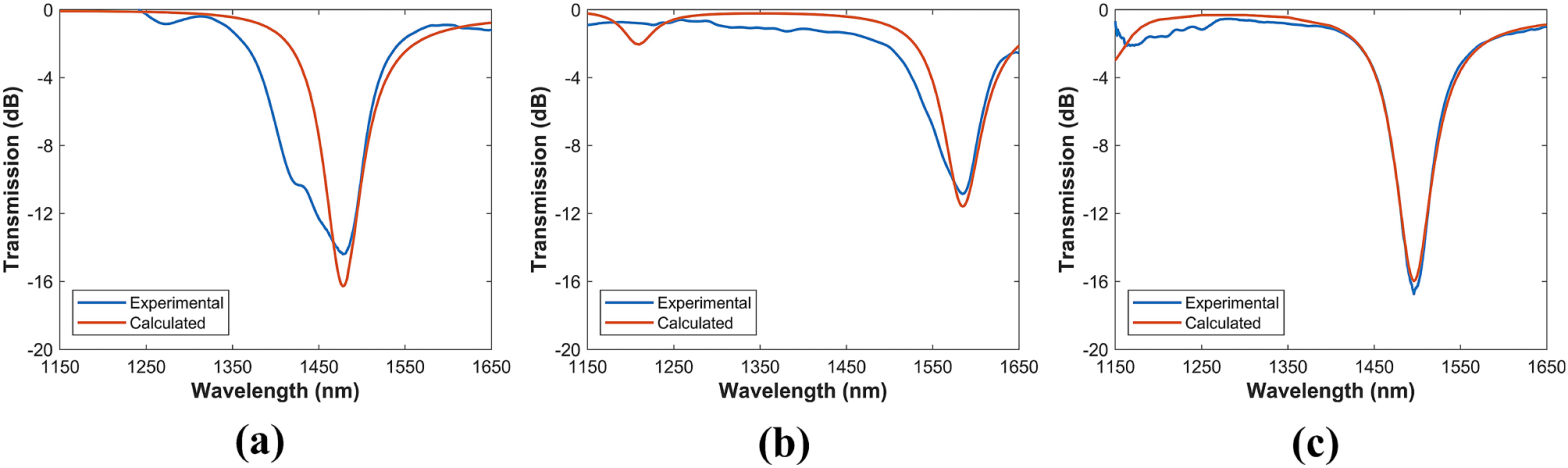
Transmission spectra of the coated D-shaped fibers. (**a**) 3-layer 1DPC, (**b**) 5-layer 1DPC, and (**c**) 6-layer 1DPC. The blue and red lines represent the experimental and the calculated spectra, respectively. The external RI is 1.3325 (water).

After the 1DPCs were fabricated, the core-guided light transmission spectra were obtained as detailed in the “Materials and methods” section. The transmitted power is presented in Fig. 4 for the three multilayer structures when the surrounding medium is deionized ultrapure water (refractive index of 1.3325 at 1500 nm), measured with a refractometer 30GS from Mettler Toledo Inc. Similarly, each device was analyzed numerically. Both numerical and experimental results agree. As it was indicated previously, the generation of these resonances can be explained by analyzing the dispersion curves in Fig. 2. Approximately at 1478, 1585 and 1496 nm, the surface modes BSW1, BSW2 and BSW3 of each structure are excited by the evanescent field of the core-guided mode in a relatively narrow wavelength range, which together with the moderate losses induced by the multilayer metal oxides, consequently cause in the experimental transmission spectrum an attenuation peak in the BSW spectral range. It is worth noting that the experimental results reveal that the effect of the BSW3 surface mode in the case of the 3-layer stack is much greater than that predicted by the simulations and consequently the attenuation peak is wider. This is confirmed by measuring the full width at half maximum (FWHM) of the experimental attenuation peaks, which are 104.5 nm, 73.5 nm, and 52.5 nm, for 3-, 5- and 6-layer structures. Also, as more layers are deposited, the spectral width of TE-BSW resonances tend to be narrower.

### Characterization of the devices as refractometers

**Figure 5:**
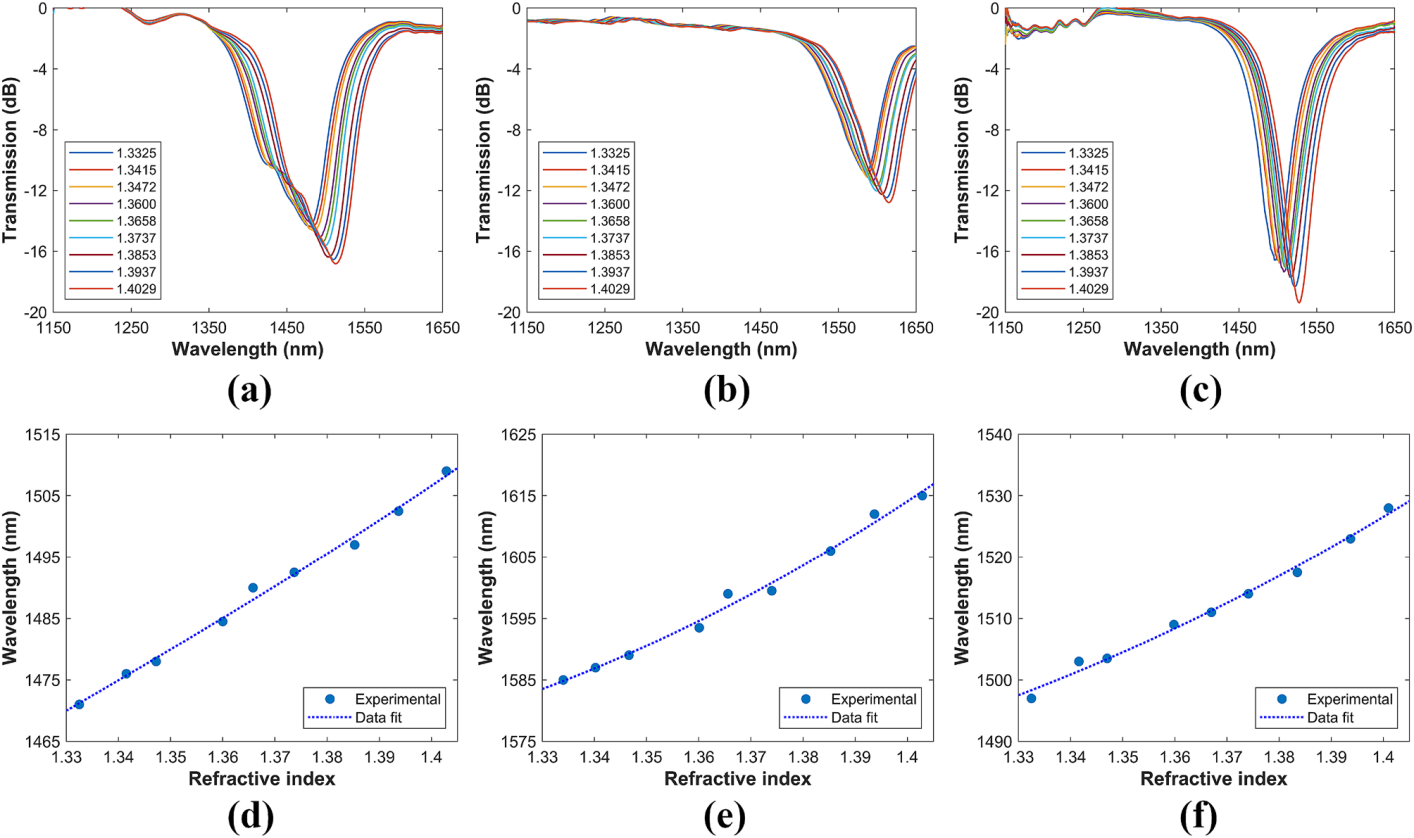
Characterization of the devices as refractometers. Experimental transmission spectra as function of the surrounding RI: (**a**) 3-layer, (**b**) 5-layer, and (**c**) 6-layer 1DPC. Evolution of the corresponding central wavelengths of the attenuation peaks: (**d**) 3-layer, (**e**) 5-layer and, (**f**) 6-layer 1DPC.

When the surrounding medium is changed, the evanescent field of the BSW will be affected, and, thereby, the phase matching condition will be altered^43^. In order to characterize the response of the devices presented in the previous section when they are used as refractometers, their 1DPCs were immersed in nine refractive index (glycerol-water) solutions and the generated transmission spectra were captured. These spectra, represented in the top row of Fig. 5, confirm that the BSWs generated by the $\hbox {SnO}_2$/CuO multilayers are highly sensitive to surrounding RI index variations. It can be appreciated that the BSW resonances shift to the red when they are subsequently immersed in solutions with increasing RI value. The evolution of the central wavelengths of the attenuation peaks when the surrounding RI is increased are presented in the bottom row of Fig. 5. As can be seen in these figures, the sensing response exhibits a nonlinear feature, which is due to the wavelength-dependent mode coupling between the core-guided mode and the BSW mode^32,43^.

Table 1 summarizes the refractometric response of the fabricated structures near to the surrounding RI $n_S$= 1.33 and $n_S$ =1.40. The device sensitivity is defined as $S_n = {\partial \lambda _{res}}/{\partial n_s}$, where $\lambda _{res}$ is the central wavelength of the attenuation peak. In addition, on the assumption that a 0.1 nm resolution detector is used, the sensor resolution is defined as RES = 0.1$/S_n$^58^. The fabricated refractometric devices have comparable sensitivity to some ESW-based sensing devices^59,60^, including some BSW-based structures^33,61,62^, for example, 631 nm/RIU in Ref.^61^, 285 nm/RIU in Ref.^62^ and -168 nm/RIU in Ref.^33^. On the other hand, our BSW-excitation platform has comparable sensitivity with a tapered fiber coated with a 1DPC, which reports, respectively, 650 nm/RIU and 930 nm/RIU for the TM and TE polarizations^30^.

**Table 1:** Refractometric performance of the three fabricated structures based on few-layer 1DPC deposited on D-shaped.

Surrounding RI	Sensitivity (nm/RIU)			Resolution ($\times 10^{-4}$ $\hbox {RIU}^{-1}$)		
(RIU)	3-Layer	5-Layer	6-Layer	3-Layer	5-Layer	6-Layer
1.33	491	317	322	2.04	3.15	3.11
1.40	554	555	508	1.80	1.80	1.97

## Conclusion

In summary, we have proposed and experimentally demonstrated a novel BSW excitation platform based on D-shaped optical fibers with few-layer 1DPC deposited on its flat surface using alternating thin layers of $\hbox {SnO}_2$ and CuO. The fiber–optic-based platform enhances the light-matter interaction in a strong way compared to other optical platforms, thereby decreasing the complexity and costs of manufacturing. In this platform, BSWs can be efficiently excited through the evanescent field of the core-guided fundamental mode, resulting in a more compact, lightweight and robust alternative compared to the Kretschman prism-based configuration.

To investigate the effect of the properties of the multilayer stack, 3-, 5-, and 6-layer 1DPCs were designed and manufactured. Although most of the BSW excitation platforms require a large number of layers or materials with a large difference in RI, we demonstrated that even a 3-layer stack on the fiber-based excitation platform can sustain TE-polarized BSWs using a 1DPC consisting of materials that have a small difference in RI. Furthermore, we demonstrated the suitability of this platform for measuring RI variations, paving the way for the development of chemical sensors or biosensors. The proposed platform can be improved by selecting new materials, modifying the thicknesses of the dielectric layers, or by modifying design parameters such as the depth of the polished area, which can lead to sensors with higher sensitivities and resolutions.

Finally, the simplicity of the designed 1DPC and the use of commercially available D-shaped fibers make it a structure with broad development potential for new types of all-fiber photonic devices and sensing applications.

## Materials and methods

The D-shaped fibers were supplied by the company Phoenix Photonics. They consisted of standard single mode fiber (Corning SMF-28) with a side-polished length of 10 mm, and insertion losses of 4 *dB* at RI 1.45. The D-shaped fibers were coated using DC sputtering system K675XD from Quorum Technologies, Ltd. The thin films were deposited around the fiber, including the polished region, by using two different targets of tin oxide $\hbox {SnO}_2$, and copper oxide CuO (both of 57 mm in diameter and 3 mm in thickness). The CuO target was purchased from ZhongNuo Advanced Material Technology Co, whereas the $\hbox {SnO}_2$ target from Plasmaterials, Inc. In addition, it is important to note that the tin oxide target was oxygen depleted and therefore strictly speaking SnO_2−x_, although for the sake of simplicity we call it $\hbox {SnO}_2$ throughout the article. The parameters used for the DC sputtering depositions were argon partial pressure of $6 \times 10^{-2}$ *mbar* and an intensity of 90 *mA*.

**Figure 6:**
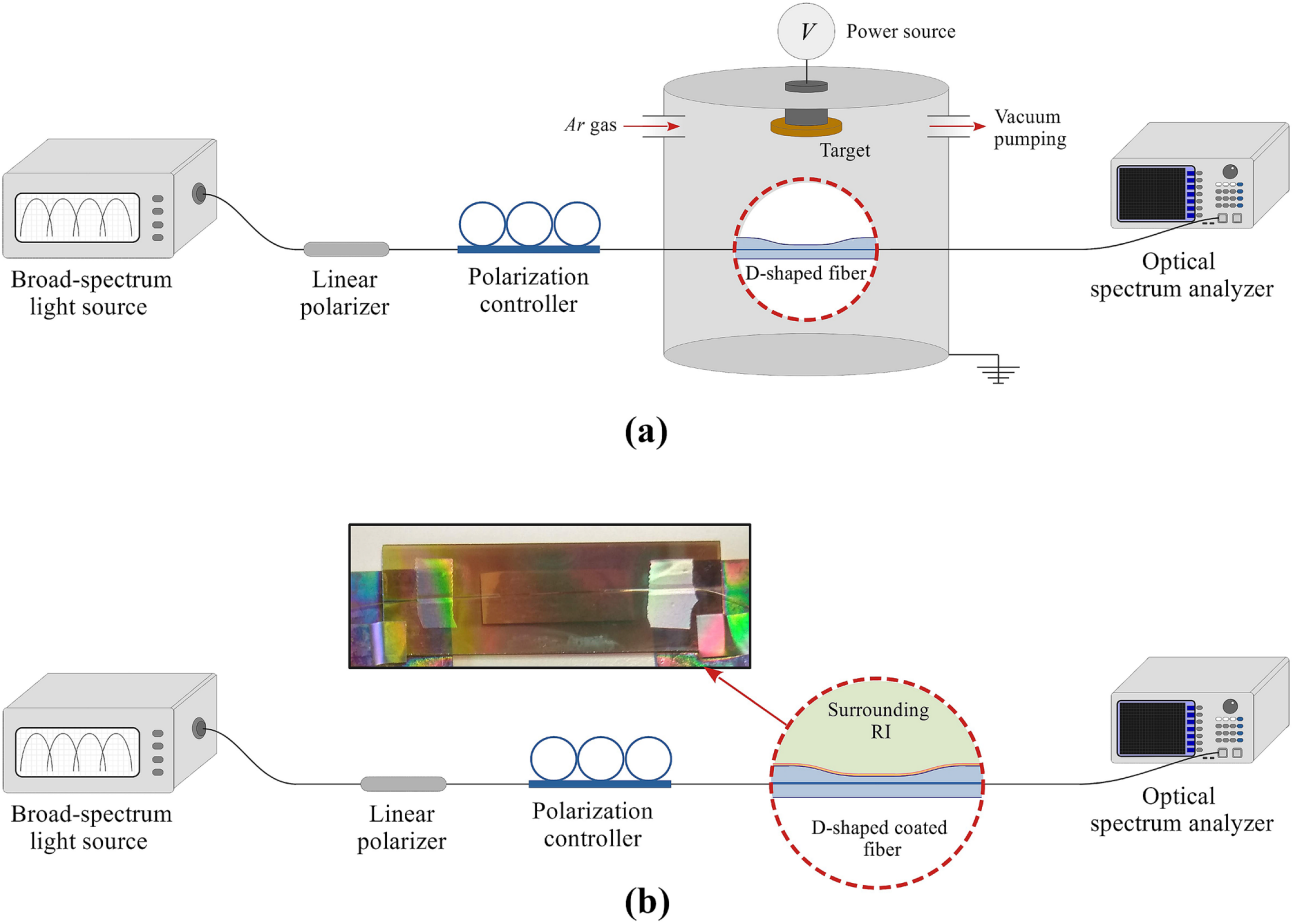
(**a**) Schematic of the experimental setup used for the thin film deposition. To observe the evolution of the optical spectrum during the thin film deposition, the pigtails connected to the polarization controller and the optical spectrum analyzer enter the sputtering machine via feedthroughs. (**b**) Schematic of the experimental setup used for the characterization of the devices as refractometers. The system allows controlling the polarization of light in order to visualize the TE resonance in the optical spectrum.

The deposition process was carried out in short time intervals and was monitored in real-time by following the spectral position of each resonance in the transmission spectra, using the setup schematized in Fig. 6a. A broad-spectrum light source Agilent 83437A (1150–1680 nm) is connected to a linear polarizer and a polarization controller. The polarized light passes to the D-shaped fiber, which is located inside the sputtering chamber, and the output is connected to an optical spectrum analyzer (OSA) Agilent 86142A. The polarization controller allows to visualize the TE resonance in the optical spectrum. The position of the optical fiber was fixed inside the sputtering machine, which included the two targets of $\hbox {SnO}_2$ and CuO, programmed so that it is deposited one thin film of $\hbox {SnO}_2$ and one thin-film of CuO alternatively and progressively. After the coating process, the fibers were removed from the sputtering machine and stored for a minimum of 48 h in air at 25$^{\circ }$C before using them for refractive index measurements. The coated fibers were characterized as refractometers using the experimental setup in Fig. 6b, for which they were immersed in refractive index solutions (glycerol-water) at different concentrations and the corresponding transmission spectra were recorded. It is worth mentioning that, to avoid distortion in the measurements, the fibers were carefully cleaned between measurements, since glycerol tends to adhere to exposed surfaces.

**Figure 7:**
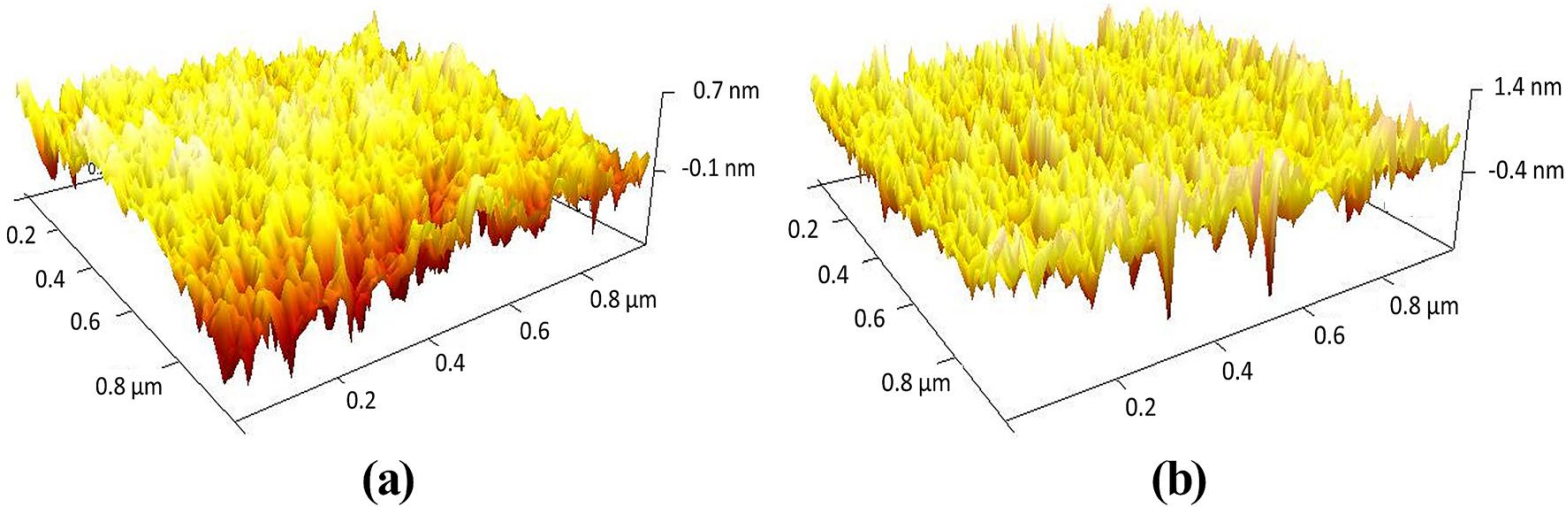
AFM microscope images showing the roughness of (**a**) the coverslip and (**b**) the flat surface of a D-shaped fiber sample (from Phoenix Photonic LTD). Homogeneous coverslip and D-shaped fiber surfaces are observed with a mean RMS roughness of 0.204 nm and 0.365 nm, respectively.

The thin films of $\hbox {SnO}_2$ and CuO deposited on coverslips where characterized with AFM microscopy (Bruker Innova with RTESPA probes in tapping mode) and with a field emission scanning electron microscope (model UltraPlus FESEM from Carl Zeiss Inc.) with an in-lens detector at 3 kV and an aperture diameter of 30 $\mu m$). The metal oxide coatings were also characterized with an ellipsometer UVISEL 2 from Horiba, with spectral range of 0.6-6.5 *eV* (190–2100 nm), an angle of incidence of 70$^{\circ }$, a spot size of 1 mm and software DeltaPsi2TM (from Horiba Scientific Thin Film Division). The thin films characterization was made on depositions over microscope glass slides, as the flat surface of the D-shaped fiber (from Phoenix Photonic LTD) has a good finish comparable to that of a coverslip. In Fig. 7, it is clear that the roughness is higher in the case of the D-shaped fiber, but it is still a subnanometric value, indicating that the results of the coverslip can be extrapolated to the D-shaped fiber.

Finally, numerical simulations were calculated using the waveguide mode solver FIMMWAVE and the optical propagation tool FIMMPROP. The simulated structure begins with the light input SMF section, which is analyzed with the finite difference method (FDM) because it is the most accurate method available for a cylindrical waveguide. In this section is adequate to find one propagating mode which corresponds to the fiber core mode. Then the light reaches the segment of the side-polished fiber on which the thin layers of $\hbox {SnO}_2$ and CuO were deposited. This segment has a much more complex geometry, so it must be analyzed using the FEM Solver, based on the finite element method. Due to the complexity of the structure, the number of modes analyzed in this section is 20 to find the surface modes that match the core mode. Finally, the light reaches the light output SMF, which is analyzed in the same way as the input section. The silica RI was calculated using the Sellmeier equation with the coefficients reported in Ref.^63^, while its extinction coefficient was neglected. In addition, the RI of $\hbox {SnO}_2$ and CuO were taken from Fig. 3e while its extinction coefficients were taken as 0.01 for $\hbox {SnO}_2$ and 0.02 for CuO^50,51,64^, to match with experimental results.

## Supplementary Information

Supplementary Information.
