# Supplementary material for: Novel Bloch wave excitation platform based on few-layer photonic crystal deposited on D-shaped optical fiber
Source: Sci Rep. 2021 May 28;11:11266. doi: 10.1038/s41598-021-90504-z (PMC8163802; doi:10.1038/s41598-021-90504-z)
Supplement: Supplementary file 1 — Supplementary Information. [file 41598_2021_90504_MOESM1_ESM.pdf]

SUPPORTING INFORMATION to the manuscript

Novel Bloch wave excitation platform based on few-layer photonic crystal deposited on D-shaped optical fiber

Esteban Gonzalez-Valencia[1,*], Ignacio Del Villar[2,3], and Pedro Torres[1]

[1]Escuela de Física, Universidad Nacional de Colombia - Sede Medellín, A.A. 3840, Medellín, Colombia,
[2]Institute of Smart Cities (ISC), Public University of Navarra, 31006 Pamplona, Spain
[3]Electrical and Electronic Engineering Department, Public University of Navarra, 31006 Pamplona, Spain
[*]egonzalv@unal.edu.co

Demonstration of surface wave coupling properties in finite one-dimensional photonic crystals

To demonstrate the coupling properties of surface waves in finite one-dimensional photonic crystals, numerical simulations were carried out using the waveguide mode solver FIMMWAVE and the optical propagation tool FIMMPROP, as detailed in the "Materials and methods" section of the main manuscript. Here, the refractive indices and extinction coefficients for SnO_2 and CuO as detailed in the main manuscript, while for TiO_2 those reported in Ref. 32 of the manuscript.

Figs. S1 and S2 present simulation results of finite photonic crystals with the same thicknesses of the structure analyzed in Fig 1a and without top layer. It shows that the field is highly enhanced at the surfaces and decays within the photonic crystal. It is evident that in a finite periodic system of alternating dielectric layers, the surface modes of each boundary can interact and become coupled in a variable degree depending both on the truncation of the outer layers and the total thickness of the photonic crystal.

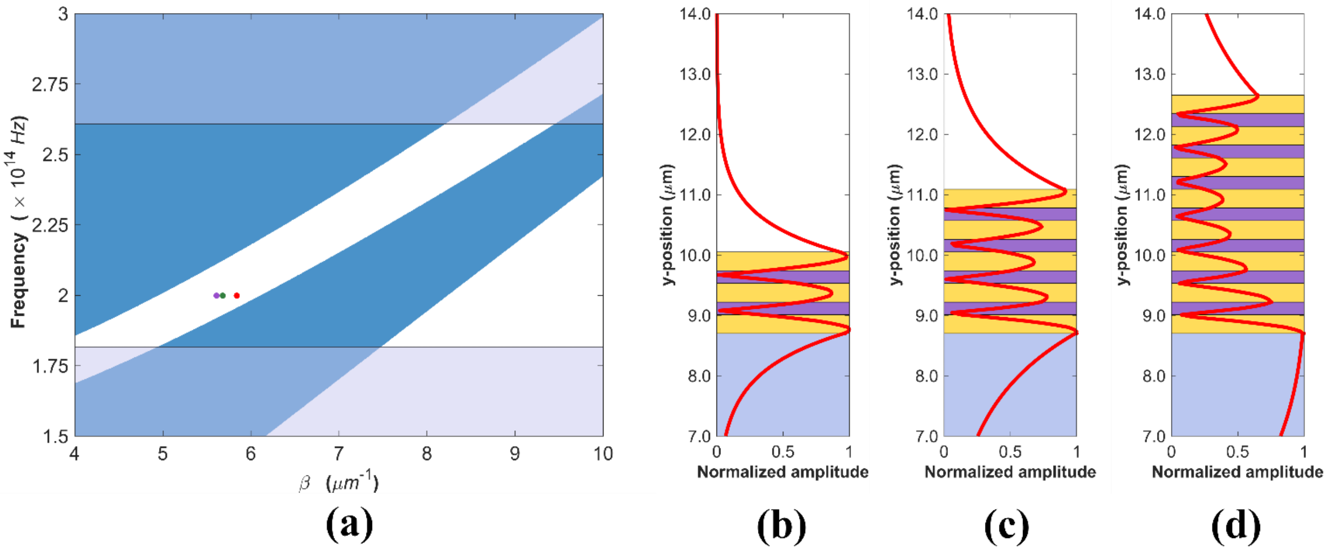

Figure S1. Coupling properties of surface waves in finite SnO_2/CuO one-dimensional photonic crystals. The thicknesses of the SnO_2 (yellow) and CuO (purple) layers are 300 and 200 nm, respectively. **(a)** Band diagram for TE polarization of the semi-infinite SnO_2/CuO 1DPC. **(b)**, **(c)**, **(d)** Electric-field intensity distributions of surface coupled modes for the 5-, 9- and 15-layer 1DPCs, corresponding to the conditions marked by the red, green and purple dots in the photonic bandgap, respectively.

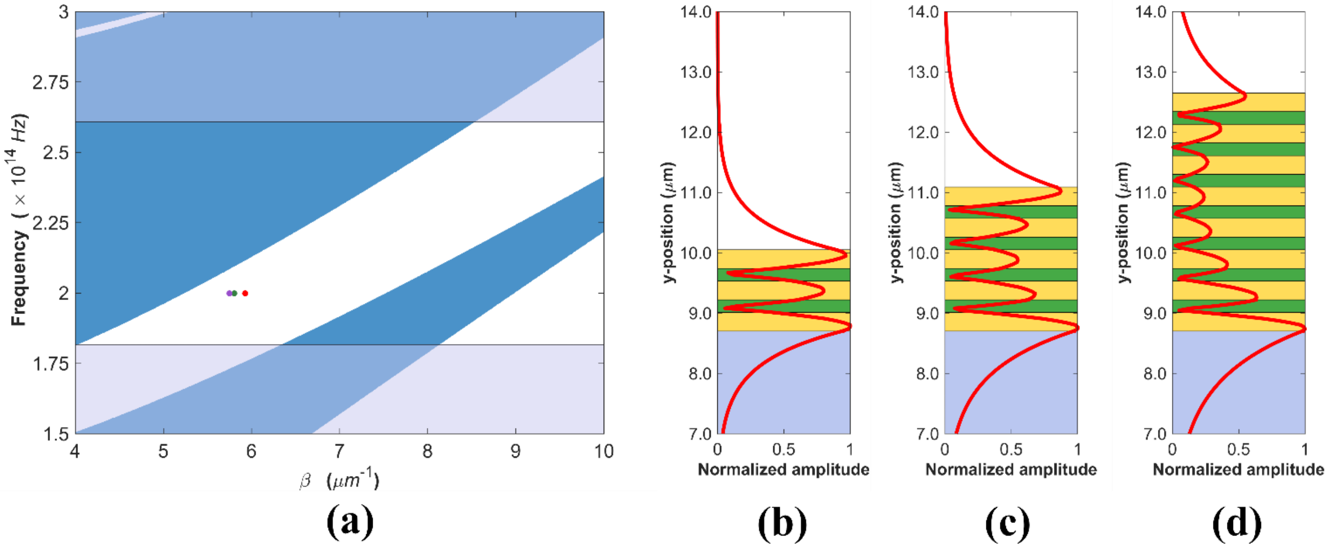

Figure S2. Coupling properties of surface waves in finite SnO_2/TiO2 one-dimensional photonic crystals. The thicknesses of the SnO_2 (yellow) and TiO_2 (green) layers are 300 and 200 nm, respectively. **(a)** Band diagram for TE polarization of the semi-infinite SnO_2/ TiO_2 1DPC. **(b)**, **(c)**, **(d)** Electric-field intensity distributions of surface coupled modes for the 5-, 9- and 15-layer 1DPCs, corresponding to the conditions marked by the red, green and purple dots in the photonic bandgap, respectively.

Influence of the higher-order BSW modes on the width of the transmission spectrum

To study the effects of excited higher-order Bloch surface waves (BSW) on the spectral response of the D-shaped optical fiber coated with the 1DPCs, numerical simulations were carried out using the waveguide mode solver FIMMWAVE and the optical propagation tool FIMMPROP, as detailed in the “Materials and methods” section of the main manuscript.

Here, three conditions for the extinction coefficients values were evaluated. For the first case, the extinction coefficients were taken as 0.01 for SnO_2 and 0.02 for CuO, in order to match with experimental results as stated in the main manuscript (Fig. S3). For the second case, the extinction coefficients were taken as 0.001 for SnO_2 and 0.002 for CuO (Fig. S4). Finally, for the third case the extinction coefficients were taken as 0.0001 for SnO_2 and 0.0002 for CuO (Fig. S5). The results of Fig. S4 and Fig. S5 complement those of Fig. S3, which confirms that the transmission spectra are mostly caused by the attenuation bands of BSW1 and BSW2 surface modes.

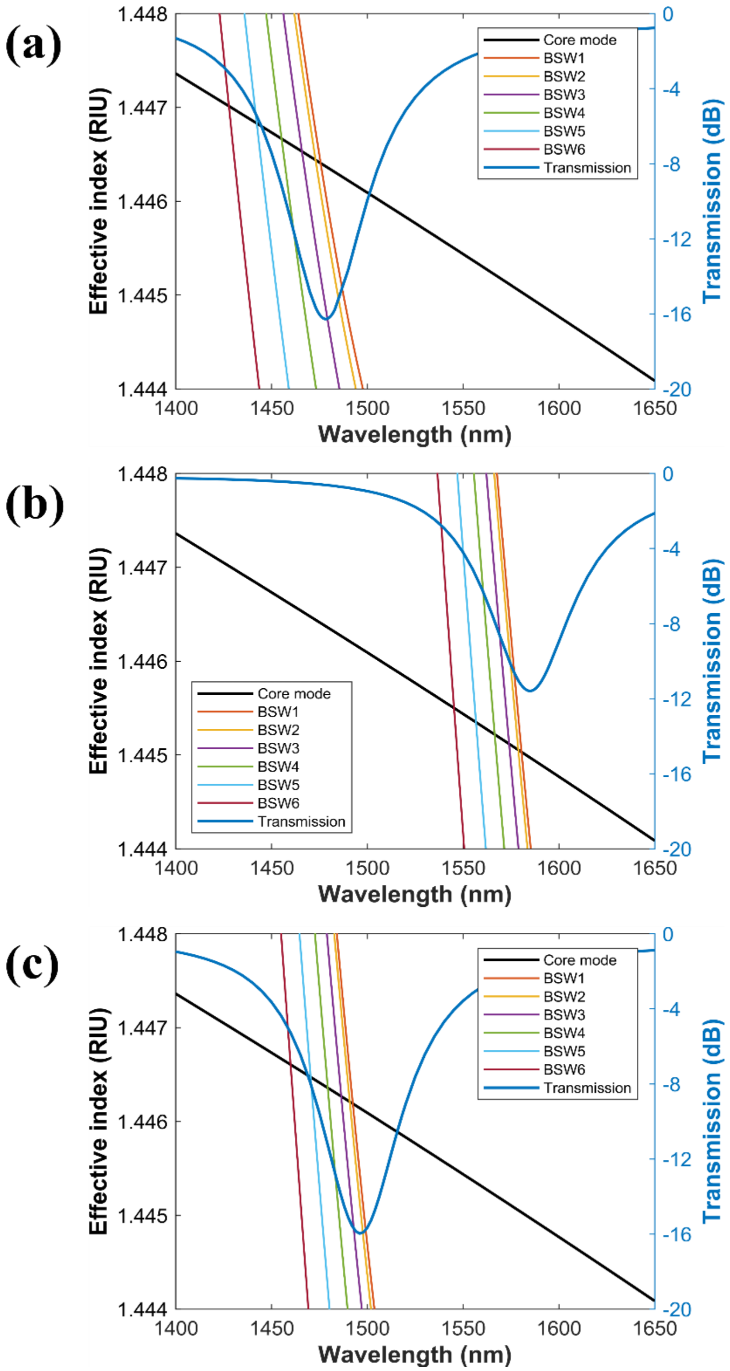

Figure S3. Dispersion curves of the core-guide mode and the BSW modes for the (a) 3-Layer, (b) 5-Layer, and (c) 6-Layer 1DPC. The blue lines (right axis) represent the calculated transmission spectra. The extinction coefficient of the SnO_2 and CuO thin films was taken as 0.01 and 0.02, respectively.

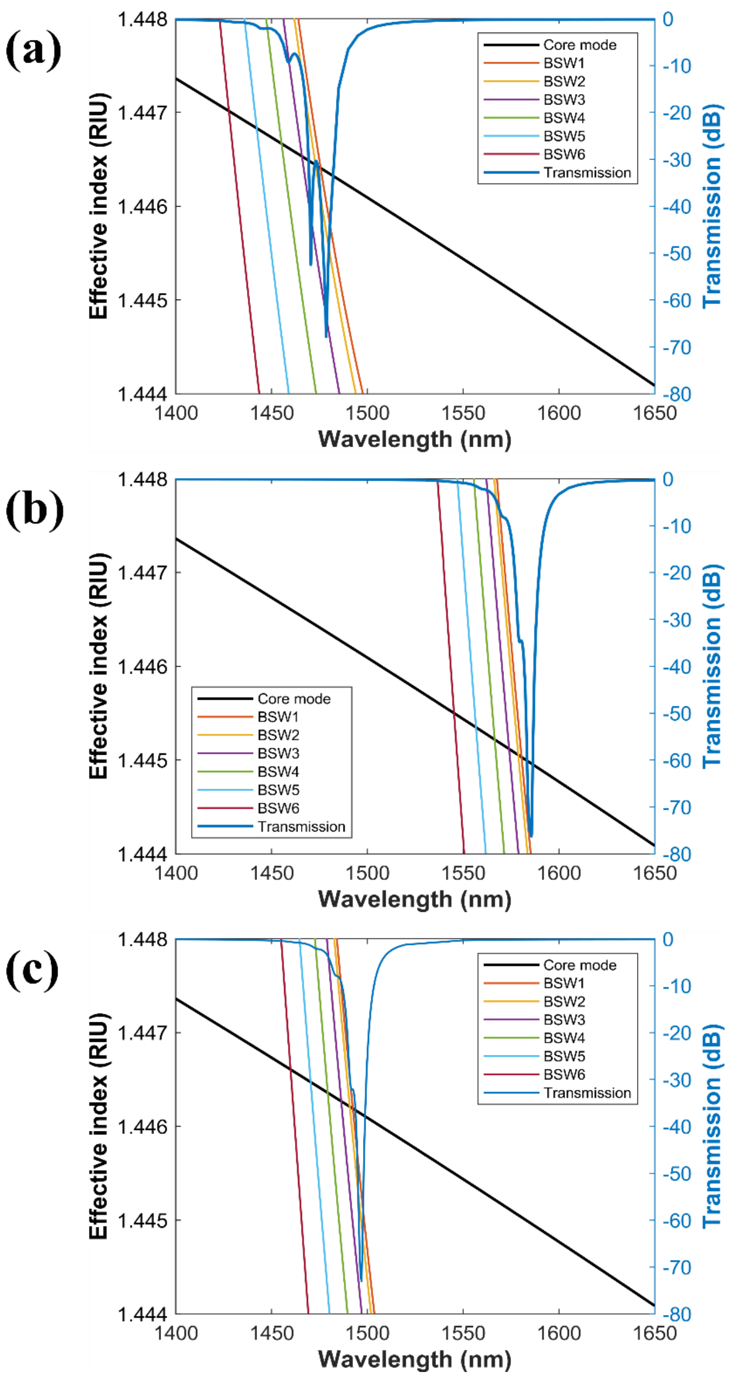

Figure S4 Dispersion curves of the core-guide mode and the BSW modes for the **(a)** 3-Layer, **(b)** 5-Layer, and **(c)** 6-Layer 1DPC. The blue lines (right axis) represent the calculated transmission spectra. The extinction coefficient of the SnO_2 and CuO thin films was taken as 0.001 and 0.002, respectively.

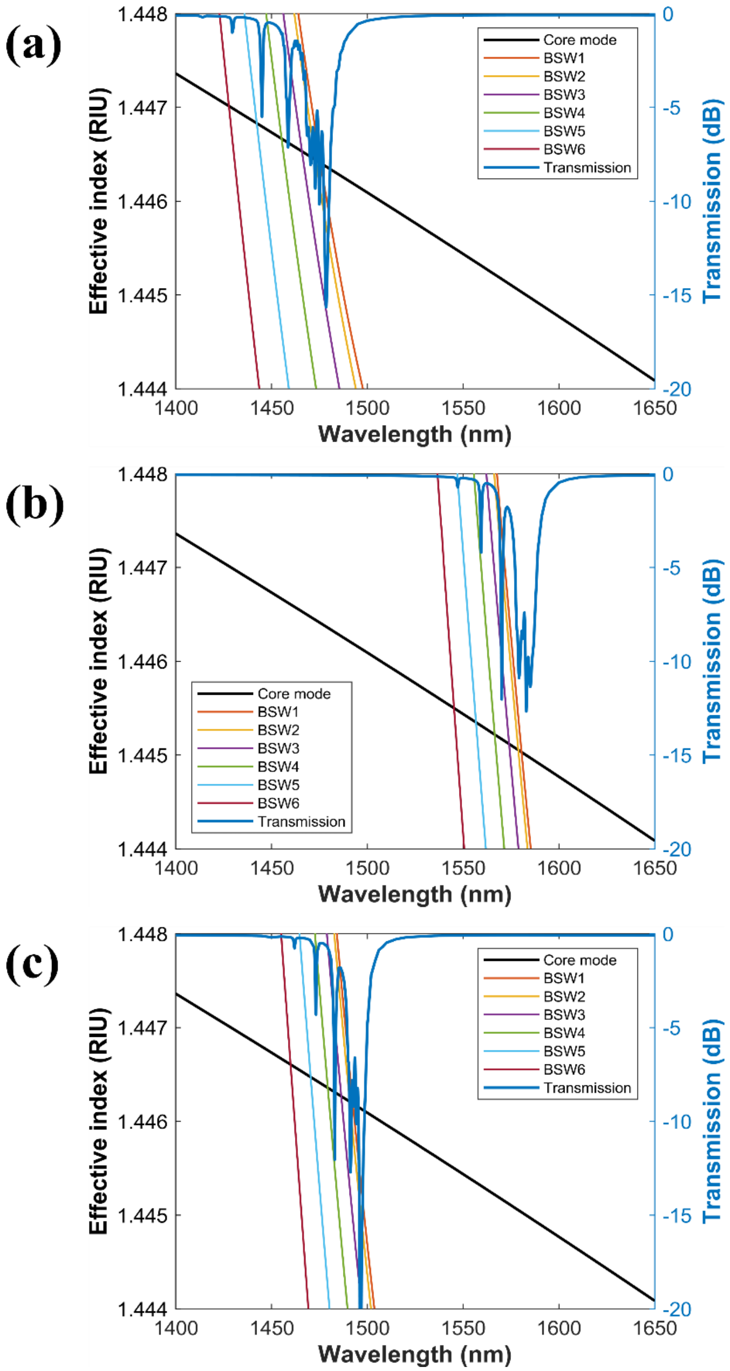

Figure S5. Dispersion curves of the core-guide mode and the BSW modes for the **(a)** 3-Layer, **(b)** 5-Layer, and **(c)** 6-Layer 1DPC. The blue lines (right axis) represent the calculated transmission spectra. The extinction coefficient of the SnO_2 and CuO thin films was taken as 0.0001 and 0.0002, respectively.
